# [Retracted] Chelerythrine chloride induces apoptosis in renal cancer HEK‑293 and SW‑839 cell lines

**DOI:** 10.3892/ol.2024.14561

**Published:** 2024-07-08

**Authors:** Xiao-Meng Chen, Meng Zhang, Peng-Li Fan, Yu-Hua Qin, Hong-Wei Zhao

Oncol Lett 11: 3917–3924, 2016; DOI: 10.3892/ol.2016.4520

Following the publication of this paper, it was drawn to the Editor's attention by a concerned reader that certain of the western blotting data shown in Fig. 4Ba on p. 3921 were strikingly similar to data that had already been published in different form in the journal *Molecular Cancer Research* several years previously, in an article written by different authors at different research institutes. Furthermore, the data shown for the p38 protein bands in Fig. 4Ba for the HEK-293 and the SW-839 cell lines were also strikingly similar, such that it seemed plausible that the same data had been included in the figure twice to represent the results from purportedly differently performed experiments.

Upon drawing these matters to the attention of the authors, they requested that the article be retracted. Therefore, owing to the fact that the contentious data in the above article had already been published prior to its submission to *Oncology Letters*, and due to a lack of confidence in the presented data, the Editor has decided that this paper should be retracted from the Journal. The Editor apologizes to the readership for any inconvenience caused.

